# The relationships of inflammatory blood markers with maternal periodontal and dental states and their effects on preterm membrane rupture development

**DOI:** 10.1186/s12903-024-04427-y

**Published:** 2024-06-04

**Authors:** Isa Temur, Katibe Tugce Temur, Safak Necati Donertas, Aycan Dal Dönertas

**Affiliations:** 1 Department of Obstetrics and Gynecology, Faculty of Medicine, Niğde Ömer Halisdemir University, Niğde, Turkey; 2 Department of Oral and Maxillofacial Radiology, Faculty of Dentistry, Niğde Ömer Halisdemir University, Niğde, Turkey; 3 Department of Periodontology, Faculty of Dentistry, Niğde Ömer Halisdemir University, Niğde, Turkey; 4 Department of Pedodontics, Faculty of Dentistry, Niğde Ömer Halisdemir University, Niğde, Turkey

**Keywords:** P-PROM, Periodontal inflammation, Dental caries, Inflammatory, Blood markers

## Abstract

**Background:**

The influence of maternal oral and dental health on the occurrence of Preterm Premature Rupture of Membranes (P-PROM) and its underlying mechanisms remain uncertain. This research seeks to investigate the impact of maternal oral and dental health on the incidence of P-PROM and its association with inflammatory markers in the blood.

**Methods:**

This study adopts a prospective case-control design methodology. The study involved 70 women diagnosed with P-PROM and delivered by an obstetrician and 79 women who had healthy deliveries with no prenatal complications. The values for DMFT (Number of decayed, missing and filled teeth) index, Gingival Index (GI), Plaque index (PI), Pocket depth (PD), Clinical attachment loss (CAL) and medical history were recorded. Mann-Whitney U test and hierarchical binomial logistic regression analysis were applied. It was considered statistically significant at *p* < 0.05.

**Results:**

The case group’s DMFT, PI, GI, PD values were statistically significantly higher than the control group (*p* < 0.001). There was no relationship between DMFT, GI, PD, CAL and inflammatory blood markers (*p* > 0.05). In the regression analysis for possible risk factors that may be effective in P-PROM, oral and dental health parameters were the most effective.

**Conclusions:**

Oral and dental health of women with P-PROM was found to be worse than that of the control group. Oral and dental health may be a potential risk factor that may contribute to adverse pregnancy outcomes associated with P-PROM.

## Introduction

Preterm premature rupture of membranes (P-PROM) is described as the rupture of fetal membranes before the 37th gestational week [1]. P-PROM is one of the causes of maternal morbidity, neonatal mortality, and Preterm labor, particularly in developing countries [2, 3]. Even though P-PROM affects only 2–5% of pregnancies, it leads to 25–30% of Preterm deliveries [4, 5]. Furthermore, there is increasing evidence that Preterm deliveries not only cause infant mortality but also increase the risk of various diseases such as respiratory distress syndrome, immunologic disorders, feeding difficulties, developmental deficiencies, and neurological disorders [6, 7]. The pathophysiological mechanism of P-PROM is multifactorial and yet not identified; however, current studies have indicated that the essential etiological mechanism of P-PROM is inflammation [8].

Periodontal disease is a long-term inflammation affecting the dental support tissues associated with dental plaque, dominated by gram-negative anaerobic microorganisms [9] Dental caries is formed through the dissolution of enamel and dentin by acidic metabolites of oral streptococci. When the cavitation formed is left untreated, oral bacteria are considered to enter blood circulation via bacterial invasion of the dental pulp [10]. During pregnancy, the complete blood count is one of the most simple, economical, and routine clinical tests [11]. The parameters obtained from complete blood count and their ratios to each other such as the neutrophil/lymphocyte ratio (NLR), platelet/lymphocyte ratio (PLR), mean platelet volume (MPV) are essential indicators of systemic inflammation [12].

There are studies suggesting that periodontitis may be associated with pregnancy complications [13–15]. Nevertheless, the possible associations of maternal oral and dental health with pregnancy complications such as P-PROM and low birth weight are controversial in the literature [16–19]. On the other hand, there are not enough studies that directly evaluate the relationship between P-PROM and maternal oral and dental health [20–23]. In this study, it was assumed that oral and dental health is worse in women with P-PROM and that it may cause changes in inflammatory blood markers.

The aim of this study is to evaluate the relationship between maternal oral health parameters and inflammatory blood markers (Monocyte/lymphocyte ratio (MLO), neutrophil/lymphocyte ratio (NLR), platelet/lymphocyte ratio (PLR), Plateletcrit (PCT), MPV/PLT and leukocyte) in women who have given birth with the diagnosis of P-PROM and to compare them with those of the control group. The null hypothesis (H0) of this study is as follows: In women giving birth with a diagnosis of P-PROM, there is no significant relationship between maternal oral health parameters and inflammatory blood markers, and these relationships do not differ statistically compared to the control group.

## Materials and methods

This study is a prospective case-control study. This study was conducted in full compliance with all versions of the Declaration of Helsinki. The study’s ethical approval was granted by Niğde Ömer Halisdemir University Faculty of Medicine Non-Interventional Ethics Committee (Decision # 2022-15).

G Power 3.0.10 (University Kiel, Germany) software was used to calculate the effect size. The effect size was calculated based on CAL data from pregnan with preterm prelabour rupture of membranes and control group in the study of Radochova et al. [20]. An effect size of 0.92 d cohen was sufficient for significance. It was calculated that at least 88 samples were required for two groups, 44 for each study group, with 0.05 type 1 error and 99% power.

The study was conducted with volunteer women who applied to Niğde Ömer Halisdemir University Faculty of Medicine Training and Research Hospital Gynecology and Obstetrics Polyclinics between March 2022 and February 2023 and underwent delivery. Women participating in the study signed the informed consent form.

The study’s case group consisted of 70 women who were diagnosed with P-PROM by a Specialist in Gynaecology and Obstetrics and who gave birth before 37 weeks’ gestation. The control group consisted of 79 women who were found by the obstetrician to have no prenatal complications and who gave birth to healthy babies at 37 weeks’ gestation or later. A total of one hundred and forty-nine women were included in the study. Women between the ages of 18 and 35 with a singleton pregnancy and no systemic disease were included in the trial.

In addition non-steroidal anti-inflammatory drugs or mouthwash for in the previous 6 months, history of periodontal treatment in the previous 3 months, systemic diseases, cases of chorioamnionitis, chronic infections, having fewer than 20 teeth or crowded teeth in the mouth, being younger than 18 years and older than 35 years, multiple pregnancies and in vitro pregnancies were exclusion criteria for the study.

P-PROM was defined as the rupture of membranes one hour or earlier than the onset of labor in pregnancies less than 37 weeks. The gestational age was determined clinically and ultrasonographically. Ruptured membranes were diagnosed by observing fluid flow through the cervix, a positive nitrazine test. The Nitrazine test is a diagnostic test used in pregnancy monitoring and is used to detect rupture of the amniotic sac (leakage of fluid). The test involves taking a sample of vaginal fluid during a speculum examination and analysing it with strips of Nitrazine dye paper. If the nitrazine strip turns blue, this indicates a possible rupture of the amniotic sac. This test is a medical tool that helps to detect amniotic fluid leakage quickly and effectively [24].

Patients enrolled in the study delivered within 24 h of having ruptured the membranes. Corticosteroids (betamethasone) were given to patients under 34 weeks’ gestation, and a single dose of antibiotics (penicillin or cephalosporin group) was given to all patients with P-PROM. Oxytocin was used to induce labour in patients who had a normal delivery. Caesarean section was performed in patients with a history of previous caesarean section. The control group received no specific treatment.

The same specialist dentist performed oral examinations of volunteer women in the case and control groups hospitalized in the ward where the delivery was performed within the first 24 h. In addition, the blood parameters were recorded through digital patient automation and from routine complete blood count data obtained by analyzing blood drawn just before delivery. Systemic inflammatory markers MLO, NLR, PLR, PCT, MPV/PLT, MLO and leukocyte values obtained from routine complete blood count were included in the study. Inflammatory blood markers were calculated based on reference studies, and these ratios had cutoff values [12, 25, 26].

Addition, obstetric history of women such as history of Preterm delivery, smoking, alcohol and coffee use, abortion history, number of births, number of pregnancies were recorded. The medicatıon given for the conditions that occur during pregnancy were recorded. In addition, data were collected on the neonate’s need for intensive care, the development of sepsis, the length of stay in intensive care and the Apgar score (The Apgar score is a method used to assess the newborn at 1 and 5 min after birth and to evaluate the response to resuscitation).

### Oral clinical examination

Patients who had given birth had an oral examination at the bedside in the Gynaecology and Obstetrics Service within the first 24 h prior to being discharged from hospital. The individuals were instructed to maintain a stationary position in their beds while oral examinations were conducted using a mirror and light source. The specialist dentist performed oral clinical examinations on women giving birth without knowing their obstetric histories.

### Dental examination

The sum of carious, missing, and filled teeth (DMFT index) were calculated according to the WHO standards (maximum 28 for DMFT ) [27].

### Periodontal examination

- Gingival Index (GI).
- Plaque index (PI).
- Probing pocket depth (PD).
- Clinical attachment loss (CAL).

In each woman, GI, PI, PD, and CAL were recorded for the entire mouth [28]. Probing measurements for PD and CAL examinations were performed at four sites. The gingival examination was performed using William’s periodontal probe (Hu- Fredy, Chicago, IL., U.S.A.). The severity of every patient’s tooth and gingival disorder was determined according to indices to assess the disease severity. Each patient’s sociodemographic data, obstetric history, delivery history, infant characteristics, and oral hygiene habits were obtained from patient follow-up forms. The data obtained from obstetric history and oral examinations were then combined and statistically analyzed.

### Statistical analysis

The software Jamovi (version 2.3.18) was used for the statistical analysis. The normal distribution was checked by Shapiro-Wilk Test. Because of the non-normal distribution, the relationship between groups was analyzed with the Mann-Whitney U test. While analyzing the categorical variables, a chi-squared test was performed. The parameters that showed significance were included in the hierarchical binomial logistic regression analysis. The optimal cutoff value for each variable in the diagnosis of periodontitis was determined with Receiver Operating Characteristic (ROC) curve analysis. The areas under the curve (AUC) were stated in a 95% confidence interval (CI). Statistically *p* < 0.05 was accepted as significant.

## Results

The study encompassed a total of 149 women, with 70 participants in the case group and 79 in the control group. There was a significant age difference between the P-PROM and control groups (*p* < 0.05). The age of those in P-PROM was significantly higher. In addition, while those with university education were more in the control group, those who were not educated were more in the P-PROM group. There was no difference between the groups in terms of obstetric characteristics, number of pregnancies, number of births and mode of delivery (*p* > 0.05). However, those who had a previous abortion and had a premature birth were significantly more in the P-PROM group (*p* > 0.05). In addition, the gestational week was significantly shorter in the P-PROM group compared to the control group, under < 37 weeks (*p* < 0.05). The range of gestational weeks in the P-PROM group was 33–36 weeks, whereas the range in the control group was 37–41 weeks. (Table 1).

**Table 1:** Characteristics of the subjects included in the study and the relationship between PPROM.

	PPROM N = 70 (47%)	Control N = 79 (53%)	p-value
Demographic characteristics			
Age	28 (22, 33) ^1^	27 (23, 29) ^1^	0.043 ^3^
Education			0.007 ^2^
None	7 (10%)	0 (0%)	
Primary education	28 (40%)	33 (42%)	
High school	22 (31%)	20 (25%)	
University	13 (19%)	26 (33%)	
**Obstetric characteristics**			
Abortion			0.002 ^2^
Yes	67 (96%)	62 (78%)	
No	3 (4.3%)	17 (22%)	
Number of pregnancies			0.062 ^2^
Primar	19 (27%)	33 (42%)	
Multipar	51 (73%)	46 (58%)	
**Gestational week mean and range**	**35,6 (33–36)** ^**1**^	**39,2 (37–41)** ^**1**^	< 0.001 ^2^
Interval with previous birth			0.201 ^2^
2 years and above	41 (59%)	38 (48%)	
< 2 years less	29 (41%)	41 (51%)	
Number of births			0.17 ^2^
Primar	22 (31%)	17 (22%)	
Multipar	48 (69%)	62 (78%)	
Type of delivery			0.29 ^2^
C/S	45 (64%)	44 (56%)	
Nvd	25 (36%)	35 (44%)	
Presence of previous premature birth			< 0.001 ^2^
Yes	18 (26%)	4 (5.1%)	
No	52 (74%)	75 (95%)	
**Newborn characteristics**			
Gender			0.46 ^2^
**Girl**	33 (47%)	42 (53%)	
**Boy**	37 (53%)	37 (47%)	
**Height (centimetres)**	49 (48, 50)	50 (49, 50)	0.001 ^3^
**Weight (grams)**	2890 (2290, 3200) ^1^	3230 (2990, 3428) ^1^	< 0.001 ^3^
**Head circumference (centimetres)**	34 (0, 37)	35 (0, 52)	0.002 ^3^
**Apgar Score**			< 0.001 ^2^
**5\6** **6\7**	**23 (33%)** **13 (18.5%)**	**0(0%)** **0(0%)**	
**7\8**	**34 (48.5%)**	**0(0%)**	
**8\9**	**0 (0%)**	**34 (43%)**	
**9\10**	**0 (0%)**	**45 (57%)**	
**NICU admission**			< 0.001 ^2^
**Yes**	**23 (32%)**	**0(0%)**	
**NIUC length of stay**			< 0.001 ^2^
**3–5 day**	**13(56%)**	**0(0%)**	
**5 and above**	**10 (44%)**	**0(0%)**	
**Sepsıs**			< 0.001 ^2^
**Yes**	**3 (13%)**	**0(0%)**	
**Respiratory Support**			< 0.001 ^2^
**Yes**	**8 (11%)**	**0(0%)**	
**Habits**			
Coffee			> 0.99 ^2^
Yes	16 (23%)	18 (23%)	
No	54 (77%)	61 (77%)	
Cigarette/Alcohol			0.81 ^2^
Yes	7 (10%)	7 (8.9%)	
No	63 (90%)	72 (91%)	
**Oral Habits**			
Brushing teeth			0.87 ^2^
Seldom	31 (44%)	35 (44%)	
1 in a day	27 (39%)	28 (35%)	
2 in a day	12 (17%)	16 (20%)	
Other oral hygiene habits			0.054 ^2^
Yes	10 (14%)	4 (5.1%)	
No	60 (86%)	75 (95%)	

n (%);^1^ Median (Min-Max)

^2^ Pearson’s Chi-squared test

^3^ Mann-Whitney U test

There was no difference between the groups in terms of smoking, alcohol consumption and coffee drinking habits and oral healthy (*p* > 0.05). There was no significant difference in gender factor in terms of newborns characteristics (*p* > 0.05). However, the newborns in the control group had significantly higher height, weight, and head circumference (*p* < 0.05). In addition, 23 (32%) of the newborns in the P-PROM group were admitted to the neonatal intensive care unit (NICU), 3 (13%) developed sepsis, 8 (11%) required ventilatory support, and Apgar scores of 5 and 6 per minute were observed in 23 (33%) of the cases and 13 (56%) remained in NICU for 3 to 5 days (*p* < 0.05) (Table 1).

Leukocyte values of individuals in the P-PROM group were significantly higher (*p* < 0.05) (Table 2). In terms of oral indexes, DMFT, PI, GI, PD and CAL values were significantly higher in the P-PROM group (*p* < 0.05). (Table 3). It was determined that there was no relationship between inflammatory blood markers and oral health indexes (Table 4).

**Table 2:** Blood counts of subjects and relationship between PPROM

Blood Counts	PPROM *N* = 70 (47%)	Control *N* = 79 (53%)	*p*-value
MVP/PLT	0.043 (0.035 0.055)	0.046 (0.039 0.065)	0.1 ^1^
PLR	120 (93 140)	110 (88 142)	0.27 ^1^
Leukocyte	11,260 (8910 13,258)	9460 (8500 11,160)	**0.006** ^1^
MLO	0.34 (0.28 0.40)	0.34 (0.27 0.39)	0.58 ^1^
NLR	3.97 (3.06 4.94)	3.77 (3.13 4.40)	0.38 ^1^
MPV	10.60 (10.00 11.47)	10.90 (10.20 11.60)	0.22 ^1^
PCT	0.26 (0.22 0.31)	0.24 (0.20 0.29)	0.089 ^1^

Median (Min-Max); ^1^ Mann-Whitney U test

**Table 3:** Oral indices of subjects and relationship between PPROM

Oral Indices	PPROM *N* = 70 (47%)	Control *N* = 79 (53%)	*p*-value
DMFT	6 (3, 10)	4 (2, 7)	**0.022** ^1^
PI	7.93 (6.09, 8.51)	5.39 (4.31, 6.41)	**< 0.001** ^1^
GI	8.21 (6.23, 9.07)	5.79 (4.07, 6.91)	**< 0.001** ^1^
PD	10.86 (9.25, 11.32)	8.04 (7.11, 8.76)	**< 0.001** ^1^
CAL	0.00 (0.00, 24.0)	0.00 (0.00, 5.0)	**0.048** ^1^

Median (Min-Max); ^1^ Mann-Whitney U test

**Table 4:** Spearman’s correlation analysis compares blood counts and oral health indices

Correlation Matrix		DMFT	PI	GI	PD	CAL
MVP/PLT	Spearman’s rho	-0.01	-0.24	-0.21	-0.18	-0.2
	p-value	0.864	0.003	0.012	0.032	0.017
PLR	Spearman’s rho	0.07	0.21	0.2	0.15	0.22
	p-value	0.418	0.009	0.017	0.072	0.007
MLO	Spearman’s rho	-0.15	0.14	0.14	0.08	0.19
	p-value	0.062	0.079	0.092	0.323	0.019
Leukocyte	Spearman’s rho	0.06	0.17	0.14	0.18	0.13
	p-value	0.463	0.04	0.09	0.026	0.102
	p-value	0.062	0.003	0.003	0.018	0.368
MPV	Spearman’s rho	-0.05	-0.14	-0.15	-0.09	-0.25
	p-value	0.568	0.089	0.076	0.257	0.002
PCT	Spearman’s rho	0.04	0.21	0.16	0.19	0.05
	p-value	0.663	0.011	0.055	0.022	0.519

In the Hierarchical binominal logistic regression analysis, DMFT (OR = 0.9007, *p* = 0.121), PI (OR = 1.8488, *p* = 0.217), GI (OR = 0.582, *p* = 0.245), PD (OR = 0.1577, *p* < 0.001), CAL (OR = 1.0789, *p* = 0.448) were added to model 1 and the model explained 49% of the variance (R^2^_MCF_ = 0.49). Abortion (OR = 2.617, *p* = 0.448) and premature delivery (OR = 3.2562, *p* = 0.217) were added to model 2 and the explanation rate increased by 2% (R^2^_MCF_ = 0.51). Medication use (OR = 6.8536, *p* = 0.245) was added to model 3 and the explanation rate increased by 3% (R2MCF = 0.54). Age (OR = 1.01, *p* = 0.875) and education were added to model 4 explanation rate increased by 5% (R^2^_MCF_ = 0.59). PL (OR = 1, *p* = 0.83), Leukocyte (OR = 1, *p* = 0.798), were added to model 5 and 59% of the variance was explained with the last model (Table 5).

**Table 5:** Presentation of hierarchical binominal logistic regression analysis

Predictor	Estimate	SE	Z	*p*-value	Odds Ratio	Lower (95% Cl)	Upper (95% Cl)	Deviance	AIC	*R* ^2^_MCF_
Intercept ^a^	14.0407	4.2347	3.3156	< 0.001	1252594.324	311.3391	5,039,496,956			
Model 1								105.42	117.42	0.49
DMFT	-0.1046	0.0675	-1.5494	0.121	0.9007	0.7891	1.0281			
PI	0.6145	0.4983	1.2333	0.217	1.8488	0.6962	4.9093			
GI	-0.5413	0.4656	-1.1627	0.245	0.582	0.2337	1.4495			
PD	-1.8472	0.3956	-4.6687	< 0.001	0.1577	0.0726	0.3424			
CAL	0.0759	0.1001	0.7584	0.448	1.0789	0.8866	1.3128			
Model 2								101.91	117.91	0.51
Abortion (Ref: No)	0.962	1.2674	0.759	0.448	2.617	0.2183	31.377			
Premature birth (Ref: No)	1.1806	0.9569	1.2337	0.217	3.2562	0.4991	21.2451			
Model 3								94.93	112.93	0.54
**Medication use (Ref: No)**	1.9248	0.7991	2.4086	0.016	6.8536	1.4312	32.8189			
Model 4										
Age	0.0099	0.063	0.1578	0.875	1.01	0.8927	1.1427	84.86	110.86	0.59
Education										
Primary education*–University	0.3797	0.7596	0.4999	0.617	1.4619	0.3299	6.4789			
High school* – University	-1.7853	0.8702	-2.0516	0.04	0.1677	0.0305	0.9233			
None* – University	-14.8738	1228.3017	-0.0121	-	-	-	-			
Model 5								84.58	118.58	0.59
**Leukocyte**	0	0	0.2565	0.798	1	1	1.0001			

^a^ Represents reference level

R^2^_MCF_: McFadden’s R^2^

**Fig. 1:**
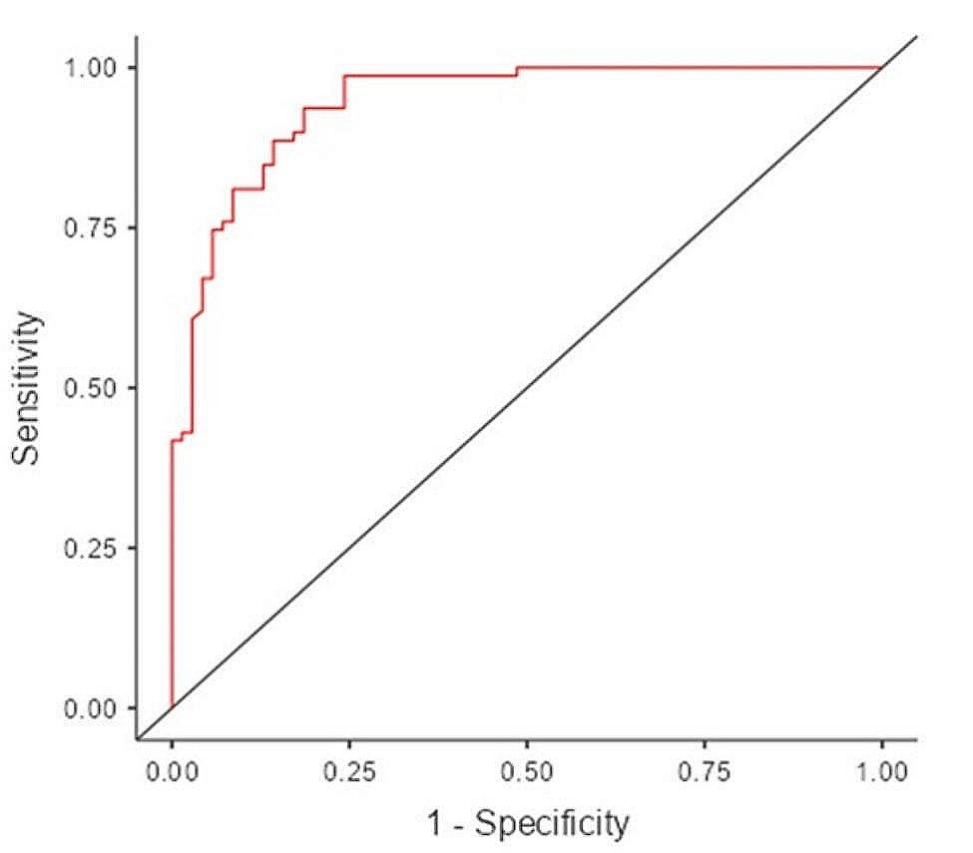
ROC curve analysis

In the ROC analysis, the cutoff value was set at 0.53, and the constructed model was seen to have 85% sensitivity and 86% specificity (Table 6; Fig. 1).

**Table 6:** Predictive Measures of the Hierarchical binominal logistic regression analysis

Accuracy	Specificity	Sensitivity	AUC
0.8523	0.8571	0.8481	0.9446

*Note * The cut-off value is set to 0.53

## Discussion

To the best of our knowledge, no study in the literature has evaluated the periodontal and dental states’ associations with inflammatory markers obtained from routine complete blood count and their probable effects on P-PROM. In addition it is not clear whether the oral and dental health of the mother is a risk factor for P-PROM and its pathogenesis. This study’s most remarkable result was that women diagnosed with P-PROM who gave birth had statistically significantly higher DMFT, PI, GI, PD values than the control group (*p* < 0.001), indicating that women diagnosed with P-PROM had worse periodontal and dental health.

When the studies investigating the maternal oral and dental health in women with P-PROM and Preterm labor were reviewed, the pioneering study was determined as the study conducted by Offenbacher et al. [29], suggesting an increased risk of premature birth due to periodontal inflammation. Furthermore, in that study, the infectious or inflammatory processes occurring in sites distal from the female genital system were also suggested to contribute to premature birth. Stadelmann et al. [23] reported that periodontal inflammation rate was higher in women diagnosed with P-PROM in their study in which they evaluated periodontal screening index, vaginal and gingival fluid collection P-PROM. In addition, although bacteria causing periodontal disease were obtained in the vagina in this study, they could not detect these bacteria in the vagina and mouth at the same time. It was reported that systemic inflammation, not the distribution of bacteria, played a role in the pathogenesis of P-PROM. Mohr et al. reported that P-PROM may develop due to systemic inflammation triggered by periodontal inflammation [21]. In this study, microbiological factors were not studied, but systemic inflammatory markers obtained from routine complete blood count were examined. However, there was no relationship between blood markers and oral and dental health parameters. Furthermore, in the study by Radochova et al. [20], in parallel with this study PI, GI, CAL and PD values of women with P-PROM were higher than in the control group. On the other hand, unlike in this study, no difference was determined between women with P-PROM and those with uncomplicated pregnancies regarding DMFT index after adjusting according to their smoking habits. In the study of Vergnes et al. [30], involving women with Preterm labor and P-PROM, the DMFT index was significantly higher in the Preterm labor group. In addition, dental caries was more common in women with Preterm labor/P-PROM. On the other hand, Wagle et al. did not find any significant association between dental caries and Preterm birth [19].

There are sociodemographic and behavioral risk factors for P-PROM. These are factors such as low socio-economic level, low education level and smoking habit [31]. BMI < 18.5 kg/m^2^, history of P-PROM or prematurity, nulliparity, multiple pregnancies, low educational level, and infections are known risk factors for P-PROM [32]. Although some factors that could be risk factors for P-PROM were excluded in this study, statistical differences were found between the case control groups in terms of factors such as age, education level, history of miscarriage and previous premature birth. As a result of the regression analysis for more than one possible risk factor, it was determined that the most effective factor was pocket depth, one of the oral and dental health parameters.

In cross-sectional studies, when patients with periodontitis, in whom periodontal diseases cause a reversible state of systemic inflammation, were compared with a healthy group regarding leukocyte [33, 34] neutrophils, increased erythrocyte sedimentation rate and serum globulin [34, 35] systemic inflammation markers were determined to be at higher levels.

The study conducted by Ustaoğlu et al. [36] found that mean platelet volume (MPV), platelet critical value (PCT) and neutrophil levels were increased in individuals with periodontal disease. However, this study did not find a significant relationship between periodontal and dental parameters and blood markers. In the study, the periodontal and dental conditions of each individual were analysed and compared on the basis of index values only, and participants were not categorised according to disease diagnosis. Measures such as GI, PI and PD play a fundamental role in the assessment of gum health. These indices provide information about the reality and prevalence of gum disease and are important in the development of treatment plans [37]. It was observed that these measurements were higher in the case group compared to the control group. This finding indicates that the gum health of the case group was worse than that of the control group and probably required more treatment.

On the other hand, various special conditions have been associated with various inflammatory blood markers [38, 39]. Toprak et al. [38] determined an association between PLR values and a state leading to P-PROM and adverse maternal and neonatal events. Low diagnostic accuracy of leukocytes in maternal serum has been reported in women with P-PROM [40]. However, Dundar et al. found no difference in leukocyte levels between women with P-PROM and the control group [39]. In this study, leukocyte values were higher in the case group. No difference was observed between other blood parameters. Even though various markers have been investigated to predict chorioamnionitis in pregnancy, none of these studies reported sufficient evidence. Furthermore, the pathophysiologic mechanisms that might explain these markers’ higher levels in pregnancy have not been fully elucidated [41, 42]. According to the current results, the higher leukocyte levels in the case group could not be associated with oral health parameters, this elevation may have increased due to a different mechanism related to P-PROM.

This study employs the use of complete blood count parameters, a commonly used and cost-effective method for assessing the systemic effects of oral health. In contrast to previous research that has focused solely on oral health, this study also explores the potential systemic effects of oral health. This approach may help in understanding the aetiology of P-PROM by providing guidance for future studies. In addition, although this study was carried out in a single center, it can give an idea about the general female population since the study was a training and research hospital.

A limitation of the study is that the oral examinations were performed at the bedside due to the special conditions of the patients. This should be taken into account when interpreting the study results. However, further studies with larger samples are needed to explain the effects of periodontal inflammation and dental disease on the pathogenesis of P-PROM. Although periodontal and dental health appear to be effective in P-PROM in this study, further studies including different systemic inflammatory markers and microbial factors are needed to explain the pathogenesis of these effects.

## Conclusion

Oral and dental health of the women with P-PROM was worse than the control group. Oral health might be one of the possible risk factors that may have adverse pregnancy outcome in terms of P-PROM.

## Data Availability

The data that support the finding of this study are available from the corresponding author upon reasonable request.

## Abbreviations

GI: Gingival Index
PI: Plaque index
PD: Probing pocket depth
CAL: Clinical attachment loss
MLO: Monocyte/lymphocyte ratio
NLR: Neutrophil/lymphocyte ratio
PLR: Platelet/lymphocyte ratio
PCT: Plateletcrit
DMFT index: Carious, missing, and filled teeth
NLR: Neutrophil/lymphocyte ratio
PLR: Platelet/lymphocyte ratio
P-PROM: Preterm premature rupture of membranes
NICU: The neonatal intensive care unit

## References

[1] Medina TM, Hill DA (2006). Preterm premature rupture of membranes: diagnosis and management. Am Fam Physician.

[2] Jena BH, Biks GA, Gete YK, Gelaye KA (2022). Incidence of preterm premature rupture of membranes and its association with inter-pregnancy interval: a prospective cohort study. Sci Rep.

[3] Lannon SM, Vanderhoeven JP, Eschenbach DA, Gravett MG, Adams Waldorf KM (2014). Synergy and interactions among biological pathways leading to preterm premature rupture of membranes. Reprod Sci.

[4] Goldenberg RL, Culhane JF, Iams JD, Romero R (2008). Epidemiology and causes of preterm birth. Lancet.

[5] Gafner M, Borovich A, Gimpel A, Peled Y, Meshulam M, Krissi H (2020). Risk factors and maternal outcomes following preterm premature rupture of membrane in the second trimester of gestation. Arch Gynecol Obstet.

[6] Kugelman A, Colin AA (2013). Late preterm infants: near term but still in a critical developmental time period. Pediatrics.

[7] Vohr B (2013). Long-term outcomes of moderately preterm, late preterm, and early term infants. Clin Perinatol.

[8] Romero R, Miranda J, Chaemsaithong P, Chaiworapongsa T, Kusanovic JP, Dong Z (2015). Sterile and microbial-associated intra-amniotic inflammation in preterm prelabor rupture of membranes. J Matern Fetal Neonatal Med.

[9] Kunnen A, van Doormaal JJ, Abbas F, Aarnoudse JG, van Pampus MG, Faas MM (2010). Periodontal disease and pre-eclampsia: a systematic review. J Clin Periodontol.

[10] Shay K (2002). Infectious complications of dental and periodontal diseases in the elderly population. Clin Infect Dis.

[11] Serrano CV, de Mattos FR, Pitta FG, Nomura CH, de Lemos J, Ramires JAF (2019). Association between Neutrophil-Lymphocyte and platelet-lymphocyte ratios and coronary artery calcification score among asymptomatic patients: data from a cross-sectional study. Mediators Inflamm.

[12] Temur I, Kucukgoz Gulec U, Paydas S, Guzel AB, Sucu M (2018). Prognostic value of pre-operative neutrophil/lymphocyte ratio, monocyte count, mean platelet volume, and platelet/lymphocyte ratio in endometrial cancer. Eur J Obstet Gynecol Reprod Biol.

[13] Cetin I, Pileri P, Villa A, Calabrese S, Ottolenghi L, Abati S (2012). Pathogenic mechanisms linking periodontal diseases with adverse pregnancy outcomes. Reproductive Sci.

[14] Moncunill-Mira J, Brunet-Llobet L, Cuadras D, Lorente-Colomé N, Pascal R, Rovira C (2021). Do the clinical criteria used to diagnose periodontitis affect the association with prematurity?. Odontology.

[15] Sgolastra F, Petrucci A, Severino M, Gatto R, Monaco A (2013). Relationship between periodontitis and pre-eclampsia: a meta-analysis. PLoS ONE.

[16] Choi SE, Choudhary A, Ahern JM, Palmer N, Barrow JR (2021). Association between maternal periodontal disease and adverse pregnancy outcomes: an analysis of claims data. Fam Pract.

[17] Caneiro L, Lopez-Carral JM, Martin-Lancharro P, Linares A, Batalla P, Blanco-Carrion J (2020). Periodontitis as a Preterm Birth Risk factor in caucasian women: a Cohort Study. Oral Health Prev Dent.

[18] Mahapatra A, Nayak R, Satpathy A, Pati BK, Mohanty R, Mohanty G (2021). Maternal periodontal status, oral inflammatory load, and systemic inflammation are associated with low infant birth weight. J Periodontol.

[19] Wagle M, D’Antonio F, Reierth E, Basnet P, Trovik TA, Orsini G (2018). Dental caries and preterm birth: a systematic review and meta-analysis. BMJ open.

[20] Radochova V, Stepan M, Kacerovska Musilova I, Slezak R, Vescicik P (2019). Association between periodontal disease and preterm prelabour rupture of membranes. J Clin Periodontol.

[21] Mohr S, Amylidi-Mohr SK, Stadelmann P, Sculean A, Persson R, Eick S (2019). Systemic inflammation in pregnant women with Periodontitis and Preterm Prelabor rupture of membranes: a prospective case-control study. Front Immunol.

[22] Lafaurie GI, Gomez LA, Montenegro DA, De Avila J, Tamayo MC, Lancheros MC (2020). Periodontal condition is associated with adverse perinatal outcomes and premature rupture of membranes in low-income pregnant women in Bogota, Colombia: a case-control study. J Matern Fetal Neonatal Med.

[23] Stadelmann PFM, Eick S, Salvi GE, Surbek D, Mohr S, Bürgin W (2015). Increased periodontal inflammation in women with preterm premature rupture of membranes. Clin Oral Investig.

[24] Muppa L, Bhavadharini K, Ramya A, Bhavadharani R (2024). Preterm Birth: a review of its early diagnosis and Prevention. J Drug Deliv Ther.

[25] Sökmen FC, Ulucaköy C (2021). Diagnostic and prognostic role of mean platelet volume and mean platelet volume/platelet ratio in patients with primary malignant bone tumor. Jt Dis Relat Surg.

[26] Uçkan K, Başkıran Y, Çeleğen İ (2022). Association of subclinical markers of inflammation with preterm premature rupture of membranes and adverse neonatal results: a case control study. Arch Gynecol Obstet.

[27] In W. World Health Organization Oral Health Surveys-Basic Methods. In. Edited by Organization WH. Geneva; 1997.

[28] Loe H (1967). The Gingival Index, the Plaque Index and the Retention Index systems. J Periodontol.

[29] Offenbacher S, Boggess KA, Murtha AP, Jared HL, Lieff S, McKaig RG (2006). Progressive periodontal disease and risk of very preterm delivery. Obstet Gynecol.

[30] Vergnes JN, Kaminski M, Lelong N, Musset AM, Sixou M, Nabet C (2011). Maternal dental caries and pre-term birth: results from the EPIPAP study. Acta Odontol Scand.

[31] Shen TT, DeFranco EA, Stamilio DM, Chang JJ, Muglia LJ (2008). A population-based study of race-specific risk for preterm premature rupture of membranes. Am J Obstet Gynecol.

[32] Bouvier D, Forest JC, Blanchon L, Bujold E, Pereira B, Bernard N et al. Risk factors and outcomes of Preterm premature rupture of membranes in a cohort of 6968 pregnant women prospectively recruited. J Clin Med 2019;8(11). 10.3390/jcm8111987 PMC6912547 31731659

[33] Shi D, Meng H, Xu L, Zhang L, Chen Z, Feng X (2008). Systemic inflammation markers in patients with aggressive periodontitis: a pilot study. J Periodontol.

[34] Botelho J, Machado V, Hussain SB, Zehra SA, Proenca L, Orlandi M (2021). Periodontitis and circulating blood cell profiles: a systematic review and meta-analysis. Exp Hematol.

[35] Franca LFC, da Silva FRP, di Lenardo D, Alves EHP, Nascimento HMS, da Silva IAT (2019). Comparative analysis of blood parameters of the erythrocyte lineage between patients with chronic periodontitis and healthy patients: results obtained from a meta-analysis. Arch Oral Biol.

[36] Ustaoglu G, Erdal E, İnanır M (2020). Does periodontitis affect mean platelet volume(MPV) and plateletcrit (PCT) levels in healthy adults?. Revista Da Associacao Med Brasileira (1992).

[37] Talebi Ardakani M, Farahi A, Mojab F, Moscowchi A, Gharazi Z (2022). Effect of an herbal mouthwash on periodontal indices in patients with plaque-induced gingivitis: a cross-over clinical trial. J Adv Periodontol Implant Dent.

[38] Toprak E, Bozkurt M, Dincgez Cakmak B, Ozcimen EE, Silahli M, Ender Yumru A (2017). Platelet-to-lymphocyte ratio: a new inflammatory marker for the diagnosis of preterm premature rupture of membranes. J Turk Ger Gynecol Assoc.

[39] Dundar B, Dincgez Cakmak B, Ozgen G, Tasgoz FN, Guclu T (2018). Platelet indices in preterm premature rupture of membranes and their relation with adverse neonatal outcomes. J Obstet Gynecol Res.

[40] Asadi N, Faraji A, Keshavarzi A, Akbarzadeh-Jahromi M, Yoosefi S (2019). Predictive value of procalcitonin, C-reactive protein, and white blood cells for chorioamnionitis among women with preterm premature rupture of membranes. Int J Gynecol Obstet.

[41] Ma M, Zhu M, Zhuo B, Li L, Chen H, Xu L (2020). Use of complete blood count for predicting preterm birth in asymptomatic pregnant women: a propensity score-matched analysis. J Clin Lab Anal.

[42] Czikk MJ, McCarthy FP, Murphy KE (2011). Chorioamnionitis: from pathogenesis to treatment. Clin Microbiol Infect.

